# Ceramides as Biomarkers and Pharmacological Targets in Heart Failure Pathophysiology

**DOI:** 10.3390/biom16040521

**Published:** 2026-04-01

**Authors:** Melania Gaggini, Cristina Vassalle

**Affiliations:** 1 Institute of Clinical Physiology, National Research Council, Via G. Moruzzi 1, 56124 Pisa, Italy; melania.gaggini@ifc.cnr.it; 2 Fondazione CNR-Regione Toscana G Monasterio, Via G. Moruzzi 1, 56124 Pisa, Italy

**Keywords:** heart failure, ceramides, lipidomic, biomarker discovery, therapeutic options

## Abstract

Heart failure (HF) is a heterogeneous condition with a prevalence of about 1–3% in the population worldwide (percentage expected to rise), related to a significant clinical and economic burden. However, despite medical advancements in HF management, difficulties in facing this condition still persist, as the etiology and phenotype largely differ between patients. Thus, the identification of further key biomarkers remains attractive. One area of interest in recent years has focused on the role of lipids in HF pathophysiology and its clinical manifestations. In this context, ceramides, complex bioactive lipids with activity in key pathways related to stress response, cellular growth, proliferation, differentiation, apoptosis, oxidative stress, inflammation, and energy production, may be important in the HF scenario, primarily for the development and application of new therapeutic strategies targeting ceramide species. Accordingly, this review aims to discuss the role of ceramides in HF pathophysiology and clinical progression in view of available evidence, focusing on the possibility of therapeutic tools for improving cardiac function and outcomes in HF by modulating ceramide signaling and metabolism.

## 1. Introduction

Heart failure (HF) is an epidemic, clinically heterogeneous condition (with a prevalence of about 1–3% of the population worldwide, actually, with this percentage expected to rise), related to a significant clinical and economic burden, because it affects patient morbidity and survival, loss of functional capacity, worsens quality of life, and elevates sanitary costs [1].

Major scientific societies in the world actually try hard to propose a consensus on a universal definition and classification of HF, about which there is still some controversy [2,3]. One of the most adopted definitions is the classification of chronic HF according to left ventricular ejection fraction (LVEF, which is based on the blood volume percentage that the left ventricle is able to eject during the systole phase), proposed in the 2021 ESC HF Guidelines, defining HF as a condition either presenting with reduced ejection fraction (HFrEF, LVEF ≤ 40%), HF with preserved ejection fraction (HFpEF, LVEF ≥ 50%), or HF with mildly reduced ejection fraction (HFmrEF, LVEF 41–49%) [4,5].

Although we have many tools and weapons against this disease (e.g., blood measurement of neurohormones and natriuretic peptides) as well as noninvasive (e.g., echocardiography and nuclear magnetic resonance) and invasive (e.g., heart catheterization and biopsy) diagnostic procedures and therapeutic options, difficulties in managing this condition still persist, as the etiology and phenotype largely differ in real life [6,7]. Thus, the comprehension of the underlying HF cellular and molecular pathways involved is critical to appropriately manage the disease in each patient and apply the adequate therapeutic options chosen based on an individualized, pathophysiology-guided treatment, fundamental in this type of condition.

One topic of interest in the last few years has focused on the role of lipids in the HF pathophysiology and clinical manifestations [7,8,9]. In particular, ceramide species among lipids have recently emerged as biomarkers of HF, with prognostic value in patient risk stratification and adverse outcome prediction [10,11,12]. Thus, further knowledge on the value of ceramides in HF also enables the development and application of new therapeutic options targeting ceramide metabolism in order to improve cardiac function and outcomes in such clinical settings.

This review aims to discuss the role of ceramides in HF pathophysiology and clinical progression in view of present evidence, focusing in particular on the possibility of options for the modulation of ceramide signaling and metabolism in HF.

## 2. Structure and Biosynthesis of Ceramides

**Figure 1:**
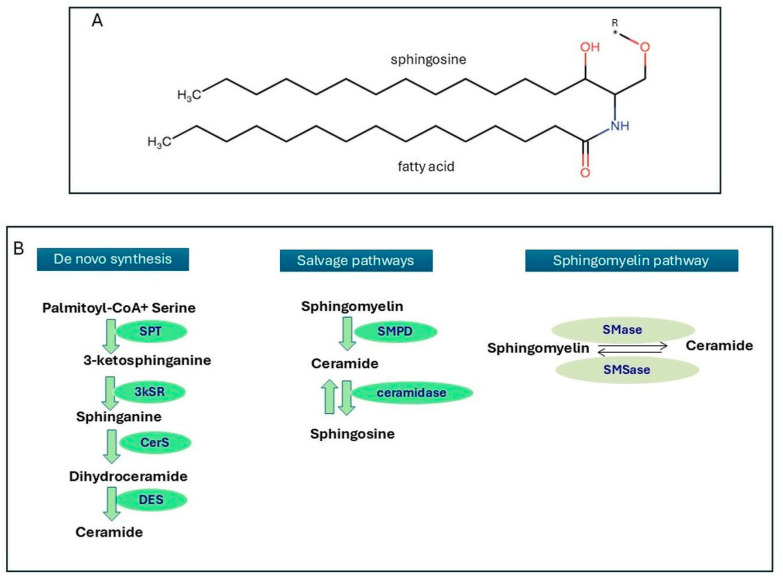
Synthesis of ceramides. (**A**) Chemical structure of ceramide. (**B**) De novo synthesis occurs in the endoplasmic reticulum (ER) by the activation of several enzymes, such as SPT: serine palmitoyl transferase; 3KSR: 3-ketosphinganine reductase; CerS: ceramide synthases; and DES: dihydroceramide desaturase. The salvage pathway is the synthesis of ceramide from sphingosine that occurs in endo/lysosomes by the action of SMPD: sphingomyelin phosphodiesterase; SMS: sphingomyelin synthases; and ceramidase. The sphingomyelin pathway is a catabolic pathway on the cellular membrane and occurs by the hydrolysis of sphingomyelin (SM) in the presence of sphingomyelinases (Smases); Smsases: sphingomyelin synthases. R* = represent the acyl chain that determines the ceramide species, which are highly diverse in chain length, hydroxylation, and saturation, particularly within the skin’s stratum corneum.

Ceramides are complex bioactive lipids whose structure is characterized by a sphingosine backbone attached to a fatty acyl chain [13]. Ceramides may differ in proportions and vary in structure (e.g., number of carbon atoms in the fatty acid, degree of saturation in terms of saturated or monounsaturated fatty acids, and length of the chains—carbon atoms—of the sphingoid backbone) (Figure 1). All these characteristics may confer different biological properties (often opposite) depending on the resulting molecule’s characteristics (e.g., length and degree of saturation) [14].

The synthesis of ceramides, which essentially takes place in the endoplasmic reticulum, is characterized by three main pathways (Figure 1):

(a) De novo synthesis: the reaction of palmitoyl-CoA and serine (catalyzed by serine palmitoyl transferase-SPT) leads to an intermediate reduced by 3-keto-dihydrosphingosine to sphinganine and then acylated (by ceramide synthases CerS1–6) to generate dihydroceramide, which finally is converted into ceramide (by dihydroceramide desaturase).
(b) The salvage pathway: sphingosine is hydrolyzed into ceramides (by ceramidase).
(c) The sphingomyelin pathway: sphingomyelin is hydrolyzed into ceramides (by sphingomyelinases—SMases).

## 3. Ceramides in Pathophysiological Processes

These molecules affect different pathophysiological key cellular pathways related to stress response, cellular growth, proliferation, differentiation, apoptosis, oxidative stress, inflammation, and energy production [14]. All these involvements in critical cellular functions render these lipid species critical in HF pathophysiology through a number of actions. Ceramides (specifically Cer(d18:1/16:0) and Cer(d18:1/18:0)) impair insulin signaling by activating protein phosphatase 2A (PP2A) or protein kinase C (PKC), which inhibits the Akt/PKB pathway, resulting in decreased glucose uptake [15]. Moreover, ceramides directly target mitochondria by lowering anti-apoptotic proteins (i.e., Bcl-2 and Bcl-xL) while increasing pro-apoptotic proteins (Bax and Bak). This leads to mitochondrial outer membrane permeabilization (MOMP) and the formation of ceramide-enriched channels, resulting in cytochrome c release, activation of caspase-3, and generation of reactive oxygen species (ROS) [16]. Ceramides also act as pro-inflammatory lipid mediators. They are frequently upregulated by inflammatory cytokines, such as TNF-α, IL-1β, and interferon-γ, establishing a positive feedback loop in which inflammation promotes ceramide production, further exacerbating cellular damage [17].

## 4. Ceramides in Heart Failure

### 4.1. Circulating Ceramides

**Figure 2:**
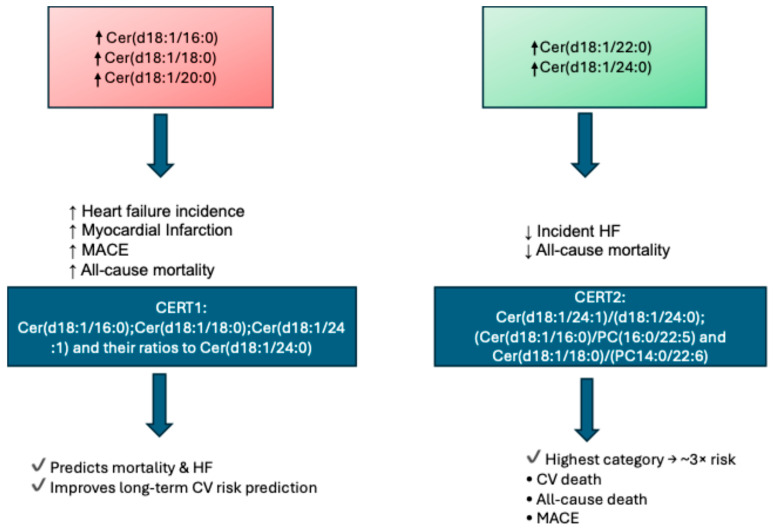
Ceramides associated with HF incidence and prognosis.

Recent studies showed that plasma ceramides are important biomarkers for HF risk prediction, mortality, and cardiovascular outcomes [18]. Long-chain ceramides, such as Cer(d18:1/16:0), Cer(d18:/:C18:0), and Cer(d18:1/20:0), are associated with HF, myocardial infarction, and cardiovascular mortality, whereas very-long-chain ceramides, such as Cer(d18:1/22:0) and Cer(d18:1/24:0), exhibit more protective effects and decreased risk of HF [19,20]. In this context, the ceramide-based risk score 1 (CERT1; obtained by measuring Cer(d18:1/16:0); Cer(d18:1/18:0); Cer(d18:1/24:1); and their ratios to Cer(d18:1/24:0)) is a tool that estimates the risk of cardiovascular disease (CVD), including mortality and HF, demonstrating prognostic utility in coronary artery disease (CAD) [21] (Figure 2). The ceramide-based risk score 2 (CERT2) is another parameter composed of four lipid ratios: one ceramide/ceramide ratio (Cer(d18:1/24:1)/(d18:1/24:0)) and two ceramide/phosphatidylcholines (PC) ratios (Cer(d18:1/16:0)/PC(16:0/22:5) and Cer(d18:1/18:0)/(PC14:0/22:6)), plus a single PC able to assess CVD risk, particularly in patients with established CAD [22]. In the COMMANDER-HF trial and the GISSI-HF trial, which enrolled patients with a history of HF to investigate the prognostic value of the CERT2 risk score, the primary outcome was CV death, but all-cause death and major adverse cardiovascular events (MACE) were also analyzed. Patients with the highest CERT2 risk category remained at almost a three-fold higher risk of CV death, all-cause death, and MACE in patients with HF [11] (Figure 2).

Specific ceramide species are involved in HF; in particular, in the PREDIMED study and the EPIC-Potsdam cohort, plasma Cer(d18:1/16:0) has been shown to be associated with a higher risk of developing HF [23]. Furthermore, in other studies, an association between circulating Cer(d18:1/16:0), Cer(d18:1/18:0), and ceramide ratios Cer(d18:1/16:0)/Cer(d18:1/24:1) and hospitalizations and mortality in patients with HF [24,25] was found. In a longitudinal study from the Cardiovascular Health Study, plasma Cer(d18:1/16:0) was positively associated with the sudden cardiac outcome [26]. Mantovani et al. showed that, in different cohort studies, plasma Cer(d18:1/16:0), Cer(d18:1/18:0), and Cer(d18:1/24:1) were associated MACE, whereas Cer(d18:1/22:0) and Cer(d18:1/24:0) had no association with cardiovascular events [27]. The ratio of plasma C24:0/C16:0 related inversely (while C16:0/C24:0 related directly) with incident HF in a meta-analysis of data from subjects in the Framingham Heart Study Offspring cohort and the large Study of Health in Pomerania (SHIP) cohort. In the same meta-analysis, each three-unit increase in the C24:0/C16:0 ratio was associated with a marked reduction in risk, corresponding to a 40% decrease in all-cause mortality. Moreover, Cer(d18:1/24:0) alone was associated with reduced all-cause mortality, whereas Cer(d18:1/16:0) alone was associated with increased all-cause mortality [19]. In another study, distinct serum ceramide species Cer(d18:1/16:0), Cer(d18:1/18:0), Cer(d18:1/20:1), Cer(d18:1/20:0), Cer(d18:1/22:1), and Cer(d18:1/24:1) were higher in HF patients when compared to controls [28]. Recently, a novel biomarker was identified based on plasma ceramides, with prognostic value for secondary events in individuals with HFpEF. In particular, a reduction in the plasma ratio C24:0/C16:0 was independently associated with all-cause mortality, CVD death, and HF hospitalization in patients with HFpEF [29] (Figure 2).

### 4.2. Tissue Ceramides

In tissue, specific ceramides, such as Cer(d18:1/16:0), Cer(d18:1/18:0), and Cer(d18:1/20:0), were increased in the myocardium of patients with HF when compared with normal controls, and they decreased after left ventricular assist device removal and improvement in left ventricular function [29]. Wiley et al. hypothesized that modulating the levels of certain sphingolipid species can influence CVD progression and alter biological processes related to HF [30]. They silenced the ceramide synthase genes (CERS) that produce 16:0 ceramide (CERS5/6) or 22:0 and 24:0 ceramide (CERS2) in immortalized human ventricular cardiomyocytes for the examination of the altered cardiac hypertrophic response to phorbol 12-myristate 13-acetate treatment by examining changes in the transcriptome [30]. The results showed that silencing CERS2 or CERS5/6 drastically altered the cardiac cell hypertrophic response, since human cardiomyocytes with silenced CERS2 appeared to have an exacerbated hypertrophy response, while cardiomyocytes with silenced CERS5/6 had a more favorable response; these findings suggested that the product metabolites deriving from these enzymes have opposing roles in the development and progression of CVD [30]. In fact, CERS2 ceramide may have a protective effect against CVD development, while CERS5/6 and 16:0 ceramide may have an adverse repercussion on CVD progression.

## 5. Interventions Targeting Ceramide Signaling and Metabolism Potentially Relevant in HF Clinical Settings

Blood ceramide levels may be modulated by different drugs (some of them commonly used in the cardiovascular field, Table 1) as well as lifestyle modifications (such as diet and exercise) [31].

### 5.1. Drugs That Regulate Ceramide Metabolism

It is well-known that statins (such as simvastatin, rosuvastatin, and proprotein convertase subtilisin/kexin type 9-PCSK 9) significantly reduce circulating ceramide concentrations, elevated in HF [32,33,34,35]. The mechanisms of statins on ceramide metabolism are not completely elucidated, but the ceramide reduction may be secondary to a broader lipid-lowering effect [28]. However, direct effects are also plausible; for example, statins directly act on the enzyme acid sphingomyelinase (ASM), blocking the oxidized low-density lipoprotein (OxLDL)-induced ASM translocation, the ceramide production, and preventing the generation of ceramide-enriched membrane rafts and the assemblage of nicotinamide adenine dinucleotide phosphate (NADPH) oxidase subunits, reducing oxidative stress (Table 1) [36,37].

Also, antidiabetic drugs, such as metformin, may affect ceramide levels in experimental and clinical studies [38,39]. In particular, one underlying mechanism has been identified, because metformin is capable of reducing ceramide synthesis, particularly in metabolic dysfunction, with the involvement of increased AMPK phosphorylation and caspase-3 activity, and as such, reducing cell death pathways related to high ceramides [40]. Moreover, metformin modulates lipid metabolism and peroxisome proliferator-activated receptors (PPARs) and sterol regulatory element-binding protein (SREBP) signaling in skeletal muscle, subcutaneous adipose and cardiac tissues, and hepatocytes [41,42,43]. Also, in this case, beyond these direct effects, the benefits of metformin might also be mediated by reduced insulin resistance or other indirect mechanisms, such as improvement in oxidative stress and inflammation, endothelial dysfunction, nonalcoholic fatty liver disease, lipid-lowering, and antihypertensive properties [44]. In particular, the relationship with inflammation and oxidative stress is of particular importance; ceramide and its derivatives (e.g., ceramide-1-phosphate, glycosyl-ceramide, sphingosine-1-phosphate, sphingomyelinase) represent intracellular signal mediators for modulation of inflammation, apoptosis, proliferation, and cell differentiation and senescence (Table 1) [45,46].

Glucagon-like peptide-1 (GLP-1) receptor agonists, a class of drugs used to treat type 2 diabetes and obesity (which act by increasing insulin and reducing glucagon secretion), modulate the ceramide profile; e.g., exenatide (10 μg twice daily) reduced levels of some adverse ceramides previously associated with cardiometabolic risk in subjects with severe obesity without diabetes (Table 1) [47,48].

More interestingly, for HF treatment, as a promising pharmacological tool in this type of patients, the sodium/glucose cotransporter 2 inhibitor (SGLT2i) empagliflozin significantly reduced the cardiac content of sphingolipids (ceramides and sphingomyelins) in Zucker diabetic fatty rats [49,50,51]. SGLT2i-treated patients (dapagliflozin 10 mg or empagliflozin 25 mg once a day for a minimum of 6 months) showed significantly lower CerC16:0, CerC22:0, and CerC24:1 levels (likely through an inhibitory action on mitochondrial function and inflammation from these drugs) (Table 1) [52].

Among beta-blockers, propranolol is an inhibitor of phosphatidic acid phosphatase (PAP; an enzyme that hydrolyzes ceramide 1-phosphate, C1P, to ceramide) [53]. In an experimental model of ischemic stroke, propranolol (a beta-blocker) demonstrated the capacity to lower ceramide levels, decreasing sympathetic nervous system activity, which in turn affects ceramide metabolism and may reduce inflammation [54]. Moreover, beta-blockers exert beneficial effects in regulating myocardial metabolism, likely reducing the production of toxic metabolites or preventing the accumulation of arrhythmogenic lipids, which include ceramides (Table 1) [55].

Angiotensin II receptor type 1 (AT1) receptor blockers, such as losartan, did not seem to affect ceramide production in an in vitro study [56]. However, in spontaneously hypertensive rats, losartan (but not hydralazine, which lowers cyclooxygenase-1 expression) reduced the endothelial expression of calcium-independent phospholipase A(2), ceramide levels, and ceramide-mediated arterial contractions (Table 1) [57]. In an experimental study, losartan modulates C16:0 levels, reducing cardiac hypertrophy [58]. Telmisartan, another AT1 receptor blocker, normalized blood ceramides in mice subjected to a high-fat diet [59].

Aldosterone has been found to be a promoter of the mineralocorticoid receptor-ceramide synthase 1 pathway required to induce C18 ceramide production, mediating cytotoxic effects and apoptosis in human umbilical vein endothelial cells, whereas eplerenone, an anti-aldosterone drug, completely blocked these adverse effects, revealing its action in ceramide metabolism (Table 1) [60].

For other drugs used in the cardiovascular and HF settings, whether direct interactions related to ceramide reduction is not fully clear, as some indirect distinct effects on adverse pathophysiological events affecting HF are more probable. In particular, currently, evidence does not support direct effects on ceramides by other drugs, such as sartans, angiotensin-converting enzyme (ACE) inhibitors, and angiotensin receptor neprilysin inhibitor (ARNI–sacubitril/valsartan), which, however, can indirectly contribute to ceramide reduction by modulating pathways that impact ceramide synthesis and metabolism. Accordingly, sartans may indirectly modulate ceramide levels through their anti-inflammatory/antioxidant effects and beneficial actions on cardiac remodeling and fibrosis (Table 1) [61].

Ceramide appears to be a mediator of Ang II/AT2–induced nuclear factor kappa-light-chain-enhancer of activated B cells (NF-κB) activation; thus, ACE inhibitors, which reduce angiotensin II, may lead to ceramide lowering, although the dimension of this effect needs further elaboration [56,62]. ARNI (sacubitril/valsartan)’s main effect in HF is to block the renin–angiotensin–aldosterone system (RAAS) and its adverse consequences that contribute to HF progression (e.g., inflammation, oxidative stress, fibrosis, and cardiac dysfunction), which can indirectly benefit lipid and ceramide metabolism [63].

Interestingly, the actual availability of drugs targeting key enzymes in ceramide metabolism represents an attractive and innovative therapeutic development, because some molecules are already used in clinical settings different from the cardiovascular one (e.g., cancer, multiple sclerosis, or neurodegenerative diseases such as Parkinson’s disease and depression), either alone or as adjuvants to traditional treatments, while others are still effectively under study, showing some promising developments [64,65]. Among the major candidates there are the six different isoforms of ceramide synthase (CERS1-6, with catalyzed ceramide production; with inhibitors such as Fumonisin B1 and fingolimod) and other enzymes such as dihydroceramide desaturase (DES1, which catalyzes the final step in the de novo ceramide biosynthesis, with the conversion of dihydroceramides to ceramides; fenretinide as one commonly known inhibitor), sphingomyelinases (SMase), which cleaves sphingomyelin to ceramide; with inhibitors AY-9944 and amitriptyline among functional inhibitors of acid sphingomyelinase, FIASMAs) (Table 1) [64].

First promising data linking these drugs and ceramide levels to ventricular dysfunction and HF are emerging. Interestingly, experimental data suggested that myriocin, a serine palmitoyltransferase (SPT, a rate-limiting enzyme in de novo ceramide production) inhibitor, reduced fatty acid oxidation and increased glucose oxidation in isolated perfused hearts, improving systolic function and survival rates [65]. Constitutive expression of cardiomyocyte Krϋppel-like factor (KLF5, which in cardiomyocytes regulates cardiac fatty acid oxidation) in KLF5 transgenic mice increases cardiac ceramide levels; treatment with myriocin prevented the increase in myocardial ceramide levels and systolic dysfunction [66]. Moreover, in HF patients, myriocin reduced ceramide accumulation in ischemic cardiomyopathy and decreased C16, C24:1, and C24 ceramides, with benefits on ventricular remodeling, fibrosis, and macrophage content following myocardial infarction [29]. Altogether, these results confirmed the role of the de novo ceramide pathway in cardiac ceramide accumulation in HF as well as the therapeutic possibility of SPT inhibition in HF settings. In this context and in view of the ubiquitous ceramide expression, the development of tissue (heart)-specific therapies is a very interesting option for translation to clinical applications. In this context, the delivery of myriocin nanoparticles would be an exciting tool to investigate for targeting ceramide de novo synthesis within atherosclerotic plaques, reducing cellular apoptosis, inflammation, and oxidative stress, and improving plaque stability in mice [67]. Moreover, attempts at RNA-based therapies (e.g., small interfering ribonucleic acid (RNA)-based approaches) or adeno-associated virus serotype 9-based strategies targeting ceramide metabolism can also be considered attractive possibilities in this field for future studies [68,69].

**Table 1:** Drug implicated in the modulation of ceramides.

Drug	Effect	Identified Mechanism for Effect on Cer	Refs.
Statins (simvastatin, rosuvastatin, and proprotein convertase subtilisin/kexin type 9-PCSK 9)	Circulating ceramide reduction	Lipid-lowering effect; modulation of ASM; oxidative stress reduction	[32,33,34,35,36,37]
Metformin	Ceramide synthesis reduction	Increased AMPK phosphorylation and caspase-3 activity; improvement of oxidative stress, inflammation, endothelial dysfunction, and nonalcoholic fatty liver disease; lipid-lowering, and antihypertensive actions	[38,39,40,41,42,43,44,45,46]
GLP-1 receptor agonist (e.g., exenatide)	Adverse ceramide reduction	Lipid-lowering effect	[47,48]
SGLT-2i (e.g., empagliflozin)	CerC16:0, CerC22:0, and CerC24:1 reduction	Modulation of mitochondrial function and inflammation	[52]
Beta-blockers (propranolol)	Circulating ceramide reduction	Inhibition of phosphatidic acid phosphatase; reduction in sympathetic nervous system activity; reduction in inflammation; modulation of myocardial metabolism	[55]
AT1 receptor blockers	Circulating ceramide reduction	Reduction in the endothelial expression of calcium-independent phospholipase A(2)	[57]
Aldosterone	Ceramide synthesis reduction	Promotion r of the mineralocorticoid receptor-ceramide synthase 1 pathway	[60]
ACE inhibitors	Circulating ceramide reduction	Reduction in angiotensin II	[61]
Fumonisin B1, fingolimod	Inhibition of ceramide metabolism enzymes	Inhibition of ceramide synthase	[64]
Fenretinide	Inhibition of ceramide metabolism enzymes	Inhibition of dihydroceramide desaturase	[64]
AY-9944 and amitriptyline	Inhibition of ceramide metabolism enzymes	Inhibition of sphingomyelinases	[64]

### 5.2. The Effects of Exercise and Diet on Ceramide Levels

Exercise training represents a key complementary tool for HF patient management, strongly recommended by current guidelines to improve functional status, exercise performance, and quality of life [70]. While acute exercise seems to increase circulating and muscular ceramide levels, regular training seems to lead to ceramide reduction; cardiorespiratory fitness (the peak oxygen uptake, oxygen consumption, VO_2_ peak) resulted in a negative association with different ceramide species [31,71]. Thus, the preventive and interventional value of exercise to improve the ceramide profile in this type of patient is expected. Nonetheless, the complexity of the relationship between exercise and ceramides, which depends on many factors (e.g., type, intensity, and duration of the physical activity performed, ceramides involved, and the type of subjects who practice exercise), renders the significance of these associations challenging to prove. However, the possibility of using physical exercise as an auxiliary tool to improve the ceramide profile, deepening the knowledge of potentially critical pathophysiological processes, and improving patient outcomes may represent an interesting research topic. In this context, experimental data suggested that the reduced expression of glucose transporter 4 (G4H) may be a key factor at the heart level as a modulator of the relationship between exercise and ceramides in HF, as exercise decreased ceramides in this model, although G4H-/- hearts failed to show reduced myocardial ceramides in response to exercise [72].

Moreover, different diet components have been found to act on ceramide metabolism (e.g., resveratrol–RSV, a polyphenol found in different plants, such as blueberries, peanuts, cranberries, and especially in red grapes and, consequently, in red wine). The chronic dysfunction within mitochondrial lipid-supported bioenergetics is reversed by RSV (200 mg (kg body weight)^−1^ for 6 weeks), preventing the development of left ventricular dysfunction associated with diabetic cardiomyopathy and normalizing total ceramide content as well as 16:0 and 18:0 ceramide species in Zucker diabetic fatty rats [73]. RSV is also a Sirtuin 1 (SIRT1) activator, SIRT1 being a regulator of antioxidant and anti-inflammatory pathways (promoting the expression of antioxidant genes as well as the inhibition of the transcription of proinflammatory genes) associated with pathophysiological events common in patients with HF and, as such, a crucial factor in the maintenance of cardiovascular homeostasis [74,75].

Other foods have been found effective in modulating the ceramide profile. Walnut consumption (48 g/day for 5 days) induced a significant reduction in harmful ceramides, together with hexosylceramides and sphingomyelins, which have been shown to mediate effects on cardiometabolic risk [76]. Higher coffee intake (>3 cups/day) was found to be associated with higher concentrations of Cer(d18:1/24:0). Cer(d18:1/24:0) in patients with chronic HF may thus warrant further investigation [77].

Interestingly, the dietary supplement alkylglycerols in a cardiac-specific transgenic dilated cardiomyopathy mouse model (presenting cardiac dysfunction, left ventricular dilation, pulmonary edema, and cardiac fibrosis) decreased ceramide species (especially those with d18:1 sphingoid backbone), which are elevated in the failing heart [78]. Conversely, experimental data suggest the accumulation of ceramides in the failing heart of rats fed with high-glucose or fast-food diets, as well as that exposure to saturated free fatty acids (FFAs) appears related to an increased de novo ceramide biosynthesis [79,80,81]. Accordingly, interesting experimental data suggest that high-palmitate feed induced ceramide and sphingomyelin accumulation in cardiac tissue, identifying a key underlying mechanism in the subsequent loss of caveolin-3 and cardiac contractile dysfunction through a defect in calcium-induced calcium release [82].

Altogether, these results open the possibility of using simple dietary interventions to change the composition and amount of ceramide, possibly modulating the outcome in HF patients. However, the multiple spectra of actions of these dietary components, together with the complexity of the ceramide network and metabolism, necessitate a better study of the overall effects of these dietary interventions in view of the type of targeted ceramide involved (which may be beneficial or adverse), dose-related effects, and primarily their interaction with other foods in a complete dietary pattern [83].

In this context, cardiac ceramide content increases in healthy and hypertrophied hearts of rats subjected to the Western diet for 9 weeks; the comparison with a model of HF (induced by doxorubicin exposure) and the Western diet showed worsened cardiac ceramides, increasing C16 and C18 content [84]. It is noteworthy that adherence to different healthy dietary patterns, such as the Mediterranean and Nordic diets, polyphenol-rich foods, and caloric restriction, has beneficial cardiovascular effects and is associated with improvement of the ceramide profile [85].

One interesting study suggests that ceramide changes could be due, at least in part, to changes in gut microbiome, as evidenced in healthy subjects where omega-3 and fiber supplementation reduced specific ceramide ratios (d18:1/16:0, d18:0/24:0, and d18:1/24:1) found to be associated with cardiovascular risk, in relation to the reduction in *Colinsella* and increases in *Bifidobacteriuim* and *Coprococcus 3* and short-chain fatty acids [86]. Thus, given the actual existing gap in the literature, knowledge on how specific foods, dietary patterns, and nutrition-related changes in gut microbiota may relate to ceramide levels in HF patients may represent an interesting area for future research.

## 6. Conclusions

Although the exact role of ceramide and related enzymes is not yet completely elucidated, these molecules without doubt have a key role in HF pathophysiology in view of the available evidence. Increasing data suggest that ceramide metabolism may be modulated in blood and at the cardiomyocyte level (e.g., by increased workload and decreased fat oxidation, which are common events during HF). Consequently, the utility of pharmacological tools, or inhibitors targeting ceramide metabolism and signaling, is surely attractive but remains challenging, whereas lifestyle modifications (e.g., diet or physical activity) could represent the most cost-effective interventional strategy to modulate ceramide metabolism. More importantly, ceramides emerge as biomarkers and targets of HFpEF, a still elusive condition, because it is characterized by heterogeneous clinical presentation with a normal ejection fraction but showing a high burden of common adverse comorbidities (e.g., aging, cardiometabolic stress, hypertension, and multiorgan fibrosis).

In this context, improvement of the most advanced techniques for ceramide measurement is highly encouraging, although the application in real clinical settings of HF will take further time. Many technical issues regarding ceramide measurements still limit their use in routine laboratories and utilization in clinical practice, including the requirement of specialized instrumentation and skilled operators, execution times, standardization and reproducibility, and costs. The safety and effectiveness of ceramide inhibitors and the identification of the correct timing of intervention still need to be clarified. One of the main problems to overcome is linked to the complexity of the ceramide system, with the possibility of a domino effect between ceramides eliciting favorable or harmful biological actions according to the ceramide targeted. In the translation from bench to clinic, the use of ceramide-based scores may be useful (e.g., CERT1), as they can help overcome the difficulties in interpreting large sphingolipid panels, making them more easily reported from laboratories and aiding the clinician’s understanding.

In the end, an exciting research area opens, which may provide advances in knowledge on the consequences derived from the actions of specific ceramides or their enzymes, as well as on the wide range of therapeutic options targeting ceramide metabolism, allowing for a more informed and personalized decision-making at various stages of HF.

## Data Availability

No new data were created or analyzed in this study.

## Abbreviations

The following abbreviations are used in this manuscript:

AT1	Angiotensin II receptor type 1
ARNI	Angiotensin receptor neprilysin inhibitor
ACE	Angiotensin-converting enzyme
Ang II/AT2	Binding of angiotensin II (Ang II) to the Type 2 (AT2) receptor
CVD	Cardiovascular disease
C1P	Ceramide 1-phosphate
CerS	Ceramide synthases
CERT1	Ceramide-based risk score
DES1	Dihydroceramide desaturase
FIASMA	Functional inhibitors of acid sphingomyelinase
GLP-1	Glucagon-like peptide-1
G4H	glucose transporter 4
HF	Heart failure
HFmrEF	HF with mildly reduced ejection fraction
HFpEF	HF with preserved ejection fraction
HFrEF	HF with reduced ejection fraction
KLF5	Krüppel-like factor 5
LVEF	Left ventricular ejection fraction
MACE	Major adverse cardiovascular events
NADPH	Nicotinamide adenine dinucleotide phosphate
NF-κB	Nuclear factor kappa-light-chain-enhancer of activated B cells
OxLDL	Oxidized low-density lipoprotein
VO_2_	Oxygen consumption
PAP	Phosphatidic acid phosphatase
PC	Phosphatidylcholines
PPARs	Proliferator-activated receptors
PCSK 9	Proprotein convertase subtilisin/kexin type 9
RAAS	Renin–angiotensin–aldosterone system
RSV	Resveratrol
RNA	Ribonucleic acid
FFAs	Saturated free fatty acids
SPT	Serine palmitoyltransferase
SIRT1	Sirtuin 1
SGLT2	Sodium/glucose cotransporter 2
SMase	Sphingomyelinases
SREBP	Sterol regulatory element-binding protein
